# Farmer and Veterinary Practices and Opinions Related to Fertility Testing and Pregnancy Diagnosis of UK Dairy Cows

**DOI:** 10.3389/fvets.2020.564209

**Published:** 2020-09-25

**Authors:** Thomas Tzelos, Natalie L. Howes, Cristina L. Esteves, Martin P. Howes, Tim J. Byrne, Alastair I. Macrae, Francesc X. Donadeu

**Affiliations:** ^1^The Roslin Institute and Royal (Dick) School of Veterinary Studies, University of Edinburgh, Edinburgh, United Kingdom; ^2^AbacusBio International Ltd., Edinburgh, United Kingdom

**Keywords:** survey, cattle, dairy, reproduction, pregnancy diagnosis, fertility tests

## Abstract

Dairy cow farming plays an important role in the UK and worldwide economies. Significant challenges are currently being faced regarding sustainability of the dairy industry. Dairy cow subfertility remains an important issue limiting herd productivity, resulting in annual losses of hundreds of millions of pounds in the UK alone. To address this, accurate monitoring of reproductive status and early detection of fertility issues in individual cows is essential. The aim of this study was to gather farmer and veterinarian opinions on current practices and perceived gaps related to diagnosis of fertility issues and pregnancy testing in UK dairy farms. Using online questionnaires, data were collected and analyzed from a total of 40 farmers and 59 veterinarians. The results showed that non-seen bulling checks and ultrasound were the most frequent tools to detect fertility issues, and that most farmers tested post-calving, and often again before or during mating. Most farmers believed that current tests did not meet their expectations, with half of those being willing to pay more than they were currently paying for fertility testing. In regard to pregnancy testing, ultrasound was most commonly used, at 30–50 days post-insemination either in individual or groups of cows. Again, most farmers believed that current tests did not meet their expectations, and a majority would consider paying a higher cost for a test that was better than those currently available. In addition, a majority of farmers would consider using a test that could detect pregnancy within 2 weeks post-insemination, if such test existed, because they believed it would help improve their herds' reproductive performance. Overall, the opinions of farmers and veterinarians indicate that there is significant scope for improving dairy herd fertility monitoring practices in the UK, through development of improved assays that can diagnose pregnancy and infertility earlier, are less disruptive to farm operations and are more cost effective than available tools. They also provide useful information to guide the future development and implementation of better diagnostics for improving reproductive performance of dairy cattle.

## Introduction

There are an estimated 120 million dairy farms worldwide holding about 300 million cows that produce 600 million tons of milk every year (1). Figures for the UK are 13,000 active dairy farmers, and 1.9 million dairy cows producing nearly 14 billion liters of milk every year. Milk production in the UK is worth £8.8 bn at wholesale level, making up almost 20% of total agricultural output (1).

A significant factor limiting productivity of the dairy industry in the UK and worldwide is cow fertility. Efficient milk production in modern dairy systems in the UK and elsewhere relies on cows being regularly calved within a 365-day interval. Given the natural 9-month gestational period in cattle, this demands a cow to be mated within the first 90 days after calving. However, based on recent figures (2, 3), calving intervals in UK dairy herds average 425 days, which is 2 months over the current industry target. This means reduced volumes of milk production, and an approximate cost of £12,000 per year for an average 100-cow farm averaging 7,000 liters milk. Moreover, infertility is the top reason for involuntary culling in dairy farms (2). Adding losses due to cows culled from failure to conceive (£13,000), the total cost of poor fertility amounts to £25,000, i.e., ~£475 million for the whole UK herd.

At the root of the issue of extended calving intervals are herd-wide conception rates, which continue to be unacceptably low (<40%) (2). Poor conception rates in modern dairy herds result from (1) reduced reproductive capacity and disease in individual cows, of genetic and managerial/nutritional origin and (2) sub-optimal reproductive management of herds including poor oestrous and/or pregnancy detection, and insemination technique.

Reproductive problems including uterine infection and anovulation commonly arise during the *post-partum* period in association with calving difficulties, metabolic disease, mastitis or lameness. Unless addressed early, these can significantly delay or prevent conception in affected cows (4). Thus, appropriate monitoring of reproductive health during the *post-partum* period is essential to maximize the number of cows that successfully become pregnant within the desired 90-day interval. Approaches used for diagnosing infertility include reproductive tract examination normally using ultrasonography, body condition assessment and behavioral (bulling) checks. However, the extent at which different methods are utilized, and their actual efficacy for actually improving reproductive efficiency in dairy herds in the UK are not known. Accurate oestrus detection is also critical for maximizing reproductive efficiency, and different technologies are available to farmers for that purpose (5).

In the context of low conception rates, timely pregnancy detection is key to maintain acceptable calving intervals in dairy herds and ensure profitable levels of milk production. At present, pregnancy status can only be accurately determined from the 4th week post-insemination, and involves the use of transrectal ultrasonography, rectal palpation or analysis of Pregnancy-Associated Glycoproteins (PAGs) or Progesterone levels in blood or milk samples (6, 7). However, most pregnancy losses occur within the first 3 weeks after insemination (8). Thus, since oestrus detection in modern dairy systems is relatively inefficient (9), many of the cows that fail to maintain their pregnancy are not identified on time for re-breeding at the first available oestrus, 21 days post-insemination, but need to wait until the following oestrus to be re-inseminated, which unnecessarily prolongs calving intervals. Efforts have been made to develop robust molecular-based tools enabling determination of pregnancy status in cows before 21 days after insemination (10, 11). We have estimated that, based on current UK practices (2), early pregnancy diagnosis could potentially save ~£7,700/year to a 100-cow farm in increased milk production alone, resulting from the reduction in calving intervals. These figures do not include additional benefits that could be accrued from reduced culling rates, and improved reproductive performance, herd productivity and animal welfare. Importantly, considerations such as sample type and timing, turnaround times for results, cost of tests, and the need for subsequent confirmatory tests, would also need to be considered before an early diagnostic test could be successfully rolled out to the wider dairy farmer community.

With the above in mind, we wished to gain an understanding of current industry practices and opinions in relation to diagnostic tools used for identification of fertility problems (i.e., those causing failure of cows to successfully conceive on time, as indicated above, excluding oestrus or pregnancy detection) and pregnancy diagnosis of dairy herds. To do this, we collected information from farmers and veterinarians to understand current diagnostic practices in UK-based dairy farms, and to reveal existing opinions on the suitability of currently available diagnostic tests and the specific areas where improvement is needed. We hoped this information could inform the development of improved strategies to reduce the effects of infertility and increase reproductive performance in dairy herds.

## Materials and Methods

A farmer- and a veterinarian-tailored questionnaires were prepared by our team's combined expertise in animal science, farm animal medicine, agribusiness consultancy and dairy farming, using SurveyGizmo (www.surveygyzmo.com). Each questionnaire (Appendix 1) included separate questions to cover participant demographics (5 and 1 questions in the farmers' and veterinarians' questionnaires, respectively), fertility testing (7 questions in both questionnaires) and pregnancy diagnosis practices (11 and 8 questions in the farmers' and veterinarians' questionnaires, respectively). Questions were structured to maximize useful information from respondents but without pre-empting or biasing their response. Questions containing multiple Likert scales [1 (strongly disagree) to 5 (strongly agree)] in succession required a minimum of 3 Likert variables to be answered before enabling progression to the next question. The questionnaires were thoroughly checked by each member of our team prior to distribution.

Online hyperlinks to each relevant questionnaire were sent by e-mail to a UK-wide, updated list of 500 farmer and 600 veterinary contacts, respectively, all active, maintained by the Dairy Herd Health and Productivity Service (DHHPS) at University of Edinburgh's Royal (Dick) School of Veterinary Studies (R(D)SVS). The DHHPS provides professional advice and testing/diagnostic services related to herd health and management to dairy farmers and veterinarian throughout the UK. This list was chosen as it provided a source of validated contacts representing the UK-wide dairy farmer and veterinary communities. Questionnaires were available for completion online from the 7th to the 28th of February 2019, following approval from the Human Ethical Review Committee at the R(D)SVS (HERC_315_19).

Upon receipt, individual questionnaires were manually inspected to ensure they contained genuine answers, i.e., provided by an actual farmer or veterinarian. Responses from three farmer questionnaires were excluded from the analyses because the respondents were based in the Republic of Ireland or Kenya, and a veterinary questionnaire was also excluded because the respondent was not a veterinarian. Questionnaires that contained only demographic information from respondents were not included in the data analyses. Questionnaire data were analyzed using SurveyGizmo to obtain number of responses, percentages and mean (± SE) score values. The “One sample proportion test” in Minitab 17 (Minitab LLC) was used to calculate 95% confidence intervals (CI) for percentages, and Chi-Square Goodness-of-Fit tests were used in a limited number of instances to establish whether differences between the proportions of respondents choosing different answers were significant (*P* < 0.05). All Figures were prepared using GraphPad 8.0 (GraphPad Software, Inc.).

## Results

### Respondent Demographic Information

**Figure 1:**
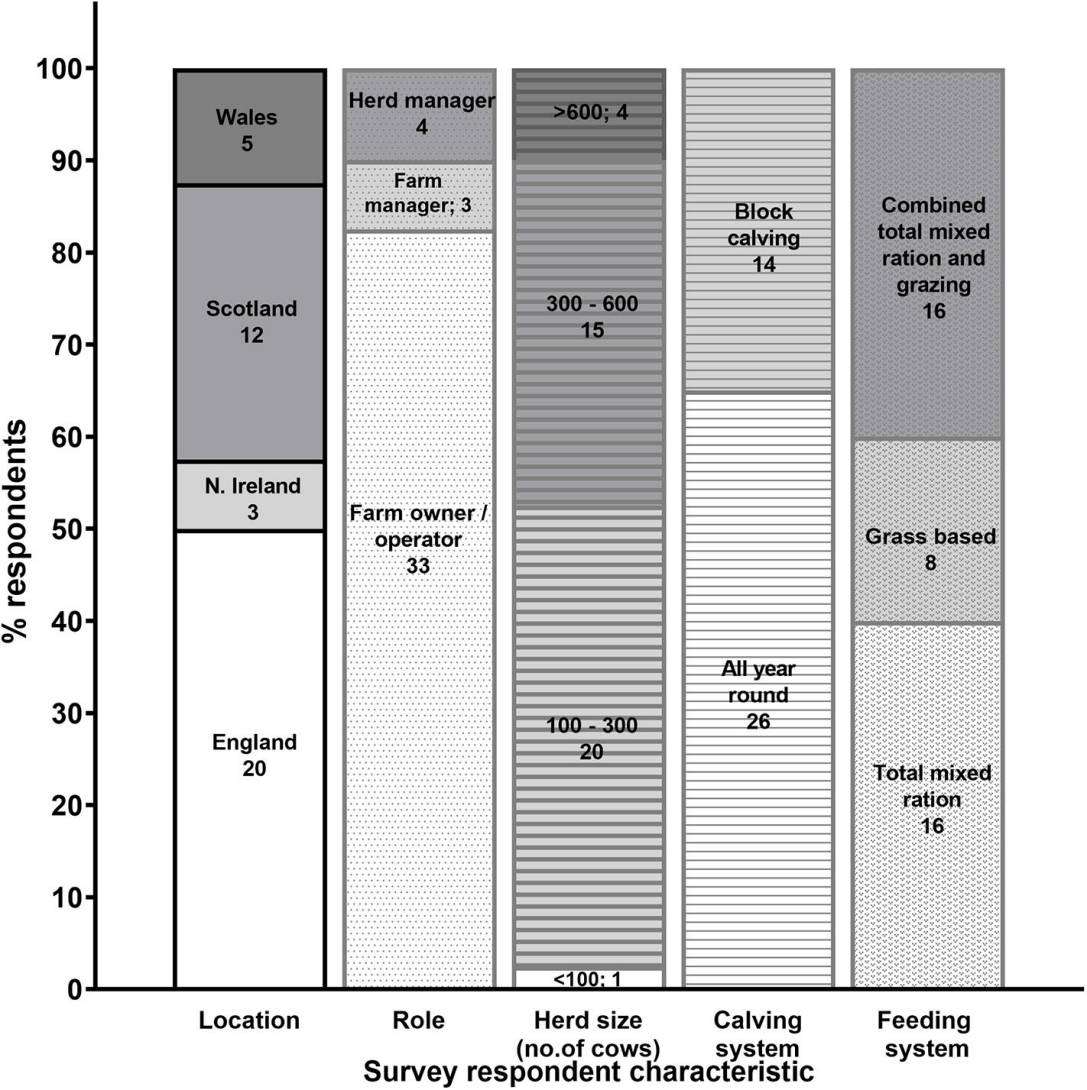
Characteristics of respondents that participated in the farmer questionnaire (*n* = 40). Numbers of respondents that selected each option for the different questions are also shown.

A total of 59 dairy farmers and 81 veterinarians participated in the questionnaires (response rates: 11.8 and 13.5%, respectively). After removing those questionnaires containing only demographic information (19 farmer and 22 veterinarian respondents), we used the responses from the remaining 40 farmers and 59 veterinarians (all of which had completed ≥65 and ≥55% of the questions in the questionnaire, respectively) for our data analyses. Farmer respondent distribution based on location, role on a farm, herd size, calving system and feeding system are shown in Figure 1. Out of the 59 veterinarians, 32 were located in England (54.2%), 13 in Scotland (22.0%), 9 in Wales (15.2%), 4 in Northern Ireland (6.8%) and 1 in Republic of Ireland (1.7%).

### Fertility Testing

**Figure 2:**
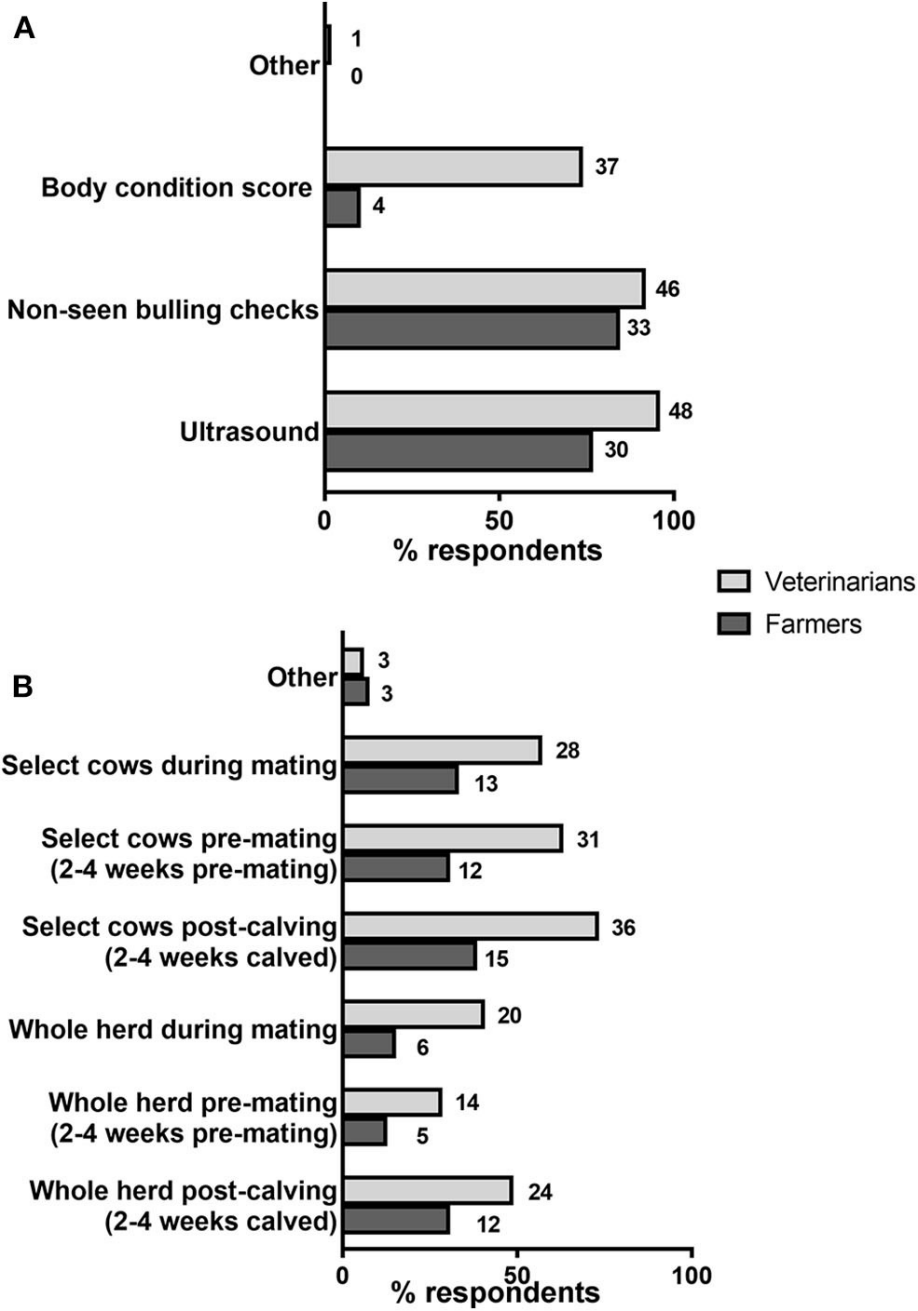
Methods and practices used for the diagnosis of fertility issues. **(A)** Percentages of farmers using/veterinarians offering each listed method are shown by horizontal bars. Number of respondents are shown next to each bar. *N* = 39 farmers, 50 veterinarians. **(B)** Percentages of respondents following each listed approach for fertility testing are shown by horizontal bars. Number of respondents are shown next to each bar. *N* = 39 farmers, 49 veterinarians.

Farmers and veterinarians were asked for the methods they were currently using or offering, respectively, to identify and/or diagnose fertility issues (Figure 2A; Table 1). The most popular approaches used by farmers were non-seen bulling checks (i.e., the absence of oestrus-related behavior; 33/39, 84.6%) and ultrasound (30/39, 76.9%; Figure 2A). A total of 14/39 farmers (35.9%) stated that they were using only one fertility test approach at their premises (Table 1), particularly either non-seen bulling checks (8/39, 20.5%) or ultrasound (6/39, 15.4%). The majority of farmers (22/39, 56.4%) were using a combination of 2 fertility tests at their premises, involving ultrasound together with either non-seen bulling checks (21/39, 53.8%) or body condition score (1/39, 2.6%). Finally, 3/39 (7.7%) farmers selected all 3 of the aforementioned fertility tests listed in the question (Table 1). As for veterinarians, all methods were widely offered by most of the respondents, with the least popular being body condition score (37/50, 70%; Figure 2A).

**Table 1:** Number of methods used to diagnose fertility issues (*N* = 39 farmers).

**Number of diagnostic methods**	**Numbers of respondents (*n*)**	**% respondents**	**% respondents (95% CI)**
1	14	35.9	21.2–52.8
2	22	56.4	39.6–72.2
3	3	7.7	1.6–20.9

The timing and form (whole herd or selected cows) in which fertility tests were carried out was another aspect that was covered in the questionnaire (Figure 2B). The responses revealed that most farmers tested post-calving, either the whole herd (12/39, 30.8%) or individual cows (15/39, 38.5%). Other farmers did not test post-calving but only before and/or during mating, either at the herd level (2/39, 5.1%) or in individual cows (6/39; 15.4%). Farmers that tested their whole herds post-calving tended to test again the whole herd before and/or during mating (7/12; 58.3%), whereas most of those that tested selected cows (rather than the whole herd) post-calving also tested individual selected cows (not the whole herd) before and/or at mating (10/15; 66.7%). It is worth mentioning that there were 2/39 (5.1%) farmer respondents who stated that they were not conducting fertility checks on their premises. In relation to the veterinarian questionnaire, a majority of respondents indicated that they provided testing in selected cows (at different times) whereas a smaller fraction were doing test at the herd-wide level, particularly post-calving and during mating (Figure 2B).

Farmers and veterinarians were then asked how much they were being charged or charging, respectively, per fertility test (Table 2). Most farmers (29/33, 87.9%) stated they paid < £5 per test, with the largest group paying < £2.00 (13/33 farmers, 39.4%). Almost half of the veterinarians (21/48, 43.8%) charged £2.00–£3.00 per test whereas very few charged ≥£5 (4/48, 8.3%).

**Table 2:** Percentage of farmers paying within each price bracket for fertility diagnosis per animal compared to what veterinary practices are charging (*N* = 33 farmers and 48 veterinarians).

**Response**	**Cohort**	**Numbers of respondents (*n*)**	**% Respondents**	**% Respondents (95% CI)**
< £2.00	Farmers	13	39.4	22.9–57.9
	Veterinarians	9	18.8	8.9–32.6
£2.00–£3.00	Farmers	8	24.2	11.1–42.3
	Veterinarians	21	43.8	29.5–58.8
£3.00–£4.00	Farmers	8	24.2	11.1–42.3
	Veterinarians	14	29.2	17.0–44.1
≥£5	Farmers	4	12.1	3.4–28.2
	Veterinarians	4	8.3	2.3–20.0

**Figure 3:**
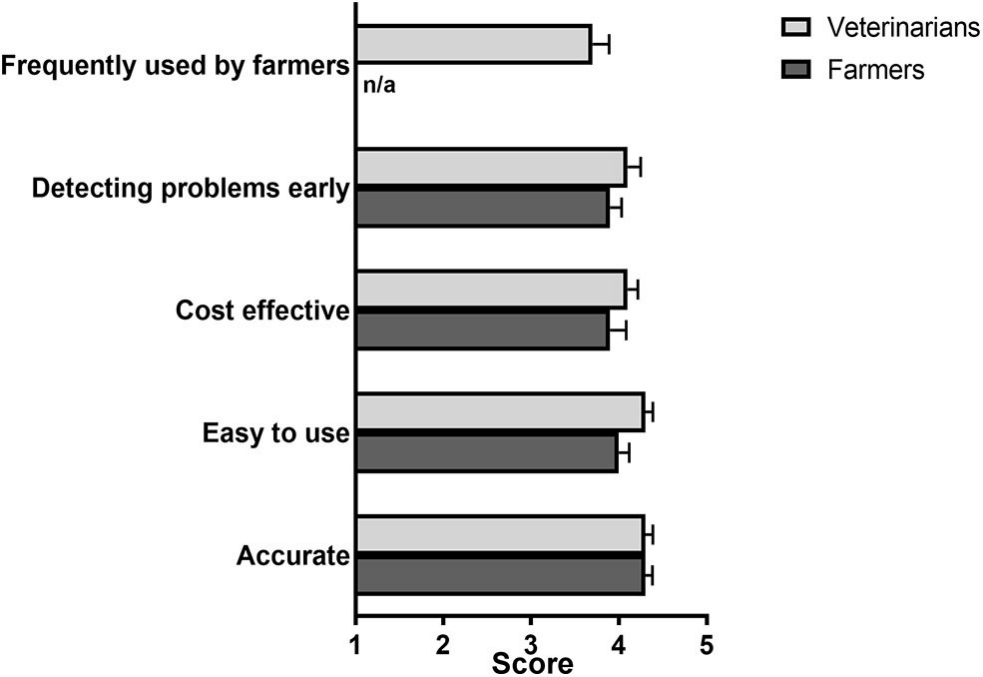
Farmer and veterinarian ratings of the characteristics of current diagnostic methods in response to the statement “The methods that I use to identify fertility issues are:” Respondents were asked to rate their agreement with each statement provided from 1 (strongly disagree) to 5 (strongly agree). Mean (± SE) scores are shown. *N* = 39 farmers, 49 veterinarians. The option “Frequently used by farmers” was only available in the veterinarian-tailored questionnaire.

The respondents were also asked whether they agreed or disagreed with different statements relating to their current approaches to fertility testing (Figure 3). In general, farmers agreed (mean scores ≥4.0) that available tests were accurate and easy to use, however they were slightly less satisfied (mean scores <4.0) with the ability to detect problems early and the cost effectiveness of fertility testing (Figure 3). Veterinarians had in general a good opinion about current methods for diagnosing fertility issues in dairy cattle (mean scores > 4.0), although they did not fully agree that current fertility diagnosis methods were frequently used by farmers (mean score, 3.7).

After rating the different characteristics of fertility test(s) (Figure 3), only a minority of farmers (5/33 or 15.2%) stated that the test(s) they were using fully met their expectations (Table 3). Of the remaining farmers, a total of 14/28 (50.0%) stated that they would pay more for a fertility test that would meet their expectations (Table 3). On the other hand, many of the veterinarians surveyed (19/49, 38.8%) were satisfied with current fertility tests (Table 3).

**Table 3:** Distribution of responses to the question “Would you be willing/do you think farmers would be willing to pay more for a fertility test that met your expectations” (*N* = 33 farmers and 49 veterinarians).

**Response**	**Cohort**	**Numbers of respondents (*n*)**	**% Respondents**	**% Respondents (95% CI)**
Yes, would pay more	Farmers	14	42.4	25.5–60.8
	Veterinarians	17	34.7	21.7–49.6
No, wouldn't pay more	Farmers	14	42.4	25.5–60.8
	Veterinarians	13	26.5	14.9–41.1
Current test meets expectations	Farmers	5	15.2	5.1–31.9
	Veterinarians	19	38.8	25.2–53.8

Farmers and veterinarians were then asked to rank their preferred sample type for a fertility test, namely, blood, urine or milk. Both groups ranked milk first followed by blood and urine in that order. Finally, they were asked their opinion on what would be an ideal timeframe for receiving/reporting results from a fertility test (Table 4). Both respondent groups overwhelmingly favored reporting of results within a 3-day frame. The largest proportion of farmers (14/34, 41.2%) stated that they would consider as acceptable 2–3 days, whereas same day reporting was the most popular option among veterinarians (23/50, 46.0%; Table 4).

**Table 4:** Farmers' and veterinarians' opinion toward the acceptable timeframe to receive fertility diagnosis results (*N* = 34 farmers and 50 veterinarians).

**Response**	**Cohort**	**Numbers of respondents (*n*)**	**% Respondents**	**% Respondents (95% CI)**
Same day	Farmers	9	26.5	12.9–44.4
	Veterinarians	23	46.0	31.8–60.7
Overnight	Farmers	10	29.4	15.1–47.5
	Veterinarians	11	22.0	11.5–36.0
2–3 days	Farmers	14	41.2	24.6–59.3
	Veterinarians	14	28.0	16.2–42.5
5–7 days	Farmers	1	2.9	0.1–15.3
	Veterinarians	2	4.0	0.5–13.7

### Pregnancy Diagnosis

**Figure 4:**
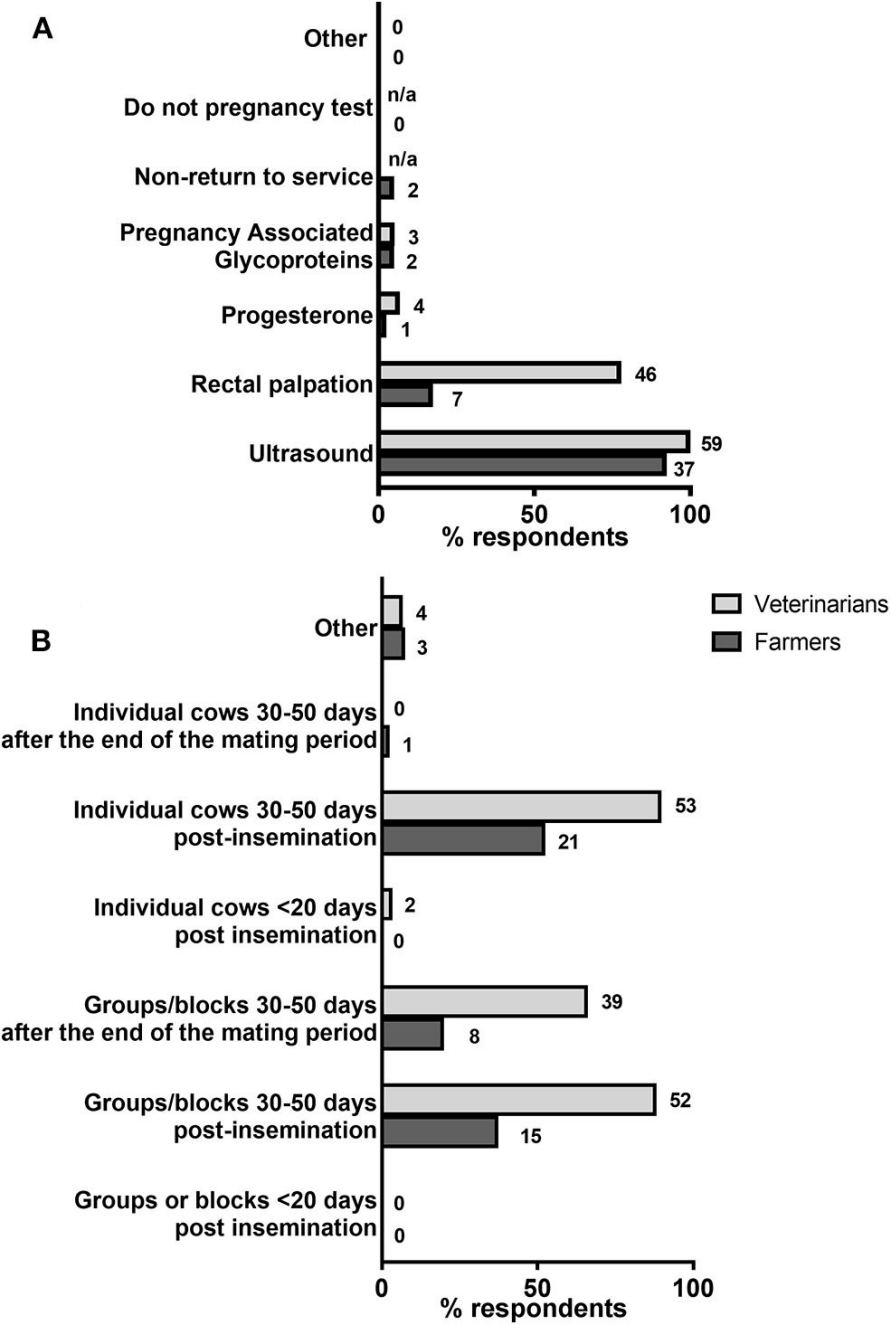
Methods and practices used for pregnancy diagnosis. *N* = 40 farmers, 59 veterinarians. **(A)** Percentages of farmers using/veterinarians offering each listed method are shown by horizontal bars. Number of respondents are shown next to each bar. The options “Non-return to service” and “Do not pregnancy test” were only available in the farmer-tailored questionnaire. **(B)** Percentages of respondents selecting each listed approach for pregnancy diagnosis are shown by horizontal bars. Number of respondents are shown next to each bar.

Both farmers and veterinarians were asked about the methods they were using/offering for pregnancy detection (Figure 4A). Ultrasound was by far the most common approach used by farmers (37/40, 92.5%), followed by rectal palpation (7/40, 17.5%). Only one farmer (2.5%) used neither of these two methods in their herd. Twenty nine of 40 (72.5%) farmers stated that ultrasound was the only method they used, and 2/40 (5.0%) that they only used rectal palpation. The remaining farmers (9/40, 22.5%) used a combination of 2 diagnostic methods. These included ultrasound together with either rectal palpation (5/40, 12.59%), non-return to service (2/40, 5.0%) or pregnancy-associated glycoproteins (1/40, 2.5%), or progesterone together with pregnancy-associated glycoproteins (1/40, 2.5%). On the other hand, veterinarians offered mostly ultrasound and rectal palpation for pregnancy diagnosis (59/59 or 100%, and 46/59 or 78.0%, respectively), with the remaining pregnancy detection methods all being offered by <10% of respondents (Figure 4A).

Respondents were also queried about their current practices regarding timing of pregnancy testing (Figure 4B). The majority of farmers stated that they normally tested cows at 30–50 days post-insemination, either individually (21/40, 52.5%) or in groups/blocks (15/40, 37.5%), whereas a third group of farmers tested only groups/blocks of cows at 30–50 days after the end of the mating season (8/40, 20.0%). Pregnancy testing at <20 days post insemination was not selected by any farmer. Moreover, among the 21 farmers that tested individual cows 30–50 days post-insemination, some (6/21, 28.6%) would also test groups/blocks of cows 30–50 days after insemination and/or the end of the mating season, whereas among the 15 farmers that tested groups/blocks 30–50 days post-insemination, 3/15 (20.0%) would also test groups/blocks of cows 30–50 days after the end of the mating season. Veterinarians largely performed pregnancy testing in individual or groups of cows 30–50 days post-insemination, and in groups/blocks 30–50 days after the end of the breeding season (Figure 4B), with 38/59 respondents (64.4%) choosing all 3 options.

When asked about the cost of pregnancy testing, a majority of farmers (28/38, 73.7%) stated that they were charged ≤ £3.0 per pregnancy diagnosis (Table 5). Most veterinarian respondents (42/59, 71.2%) charged in the range of £2.0–£4.0, with only a small proportion (4/59, 6.8%) charging ≥5.0 (Table 5).

**Table 5:** Prices paid by farmers for pregnancy diagnostic results per animal compared to the prices charged by veterinarians (*N* = 38 farmers and 59 veterinarians).

**Response**	**Cohort**	**Numbers of respondents (n)**	**% respondents**	**% respondents (95% CI)**
< £2.00	Farmers	13	34.2	19.6–51.4
	Veterinarians	13	22.0	12.3–34.7
£2.00–£3.00	Farmers	15	39.5	24.0–56.6
	Veterinarians	25	42.4	29.6–55.9
£3.00–£4.00	Farmers	5	13.2	4.4–28.1
	Veterinarians	17	28.8	17.8–42.1
≥£5	Farmers	5	13.2	4.4–28.1
	Veterinarians	4	6.8	1.9–16.5

**Figure 5:**
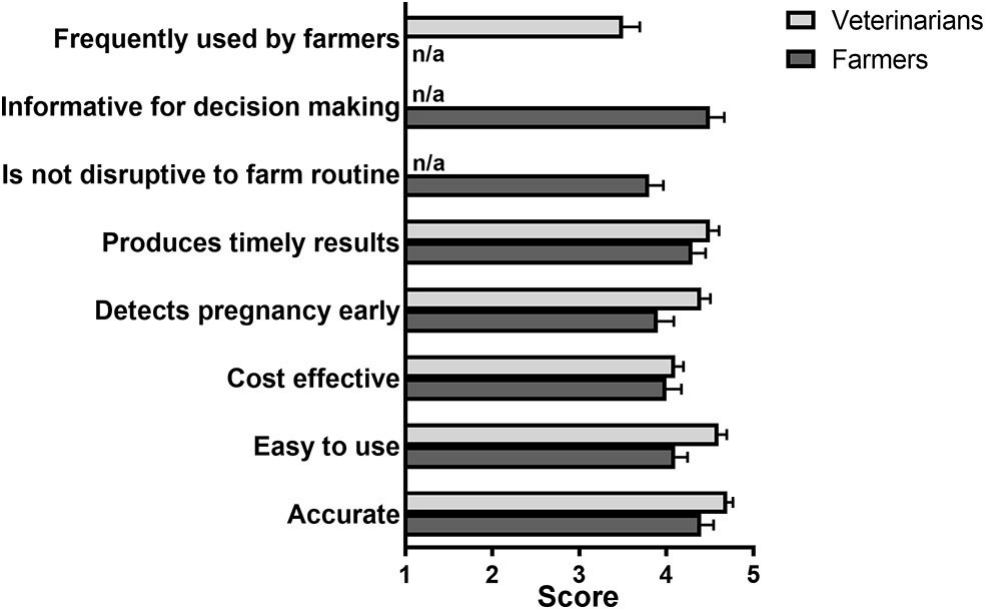
Farmer and veterinarian ratings of the characteristics of current pregnancy diagnosis methods in response to the statement “The method(s) that I use for pregnancy detection is/are...” Respondents were asked to rate their agreement with each statement provided from 1 (strongly disagree) to 5 (strongly agree). Mean (± SE) scores are shown. *N* = 40 farmers, 59 veterinarians. The options “Is not disruptive to farm routine” and “Informative for decision making” were only available in the farmer-tailored questionnaire, whilst the option “Frequently used by farmers” was only available in the veterinarian-tailored questionnaire.

The respondents were then asked for their opinion about current pregnancy diagnosis methods (Figure 5). In the farmer questionnaire, most statements were scored between 4.0 and 4.5, indicating overall satisfaction with current diagnostic methods, except for “able to detect pregnancy early” and “disruptive to farm routine” which mean scores did not reach 4.0, i.e., were below “agree.” Compared to farmer's responses, scores given by veterinarians were slightly higher (4.1–4.7), except for the statement “current pregnancy methods are frequently used by farmers” which received the lowest mean score overall (3.5; Figure 5).

Additionally, respondents' opinion was sought on whether they would consider paying a higher price if there was a pregnancy test that met all of their expectations (Table 6). A small proportion of farmers (6/40, 15.0%) stated that current tests met their expectations. Of the remaining farmers, 21/34 (61.8%) stated that they would consider a higher cost; the difference between the proportions of those that would pay more and those that would not, approached significance (*P* = 0.1). On the other hand, the majority of veterinarian respondents (38/59, 64.4%) believed that the “current test meets expectations.” Of the remaining veterinarian respondents, 5/21 (23.8%) believed that farmers would consider a higher cost to pregnancy testing, a significantly lower proportion than those that believed that farmers would not pay more for a test (*P* = 0.02; Table 6).

**Table 6:** Distribution of responses to the question “Would you be willing/do you think farmers would be willing to pay more for a pregnancy test that met your expectations” (*N* = 40 farmers and 59 veterinarians).

**Response**	**Cohort**	**Numbers of respondents (*n*)**	**% Respondents**	**% Respondents (95% CI)**
Yes, would pay more	Farmers	21	52.5	36.1–68.5
	Veterinarians	5	8.5	2.8–18.7
No, wouldn't pay more	Farmers	13	32.5	18.6–49.1
	Veterinarians	16	27.1	16.4–40.3
Current test meets expectations	Farmers	6	15.0	5.7–29.8
	Veterinarians	38	64.4	50.9–76.4

**Figure 6:**
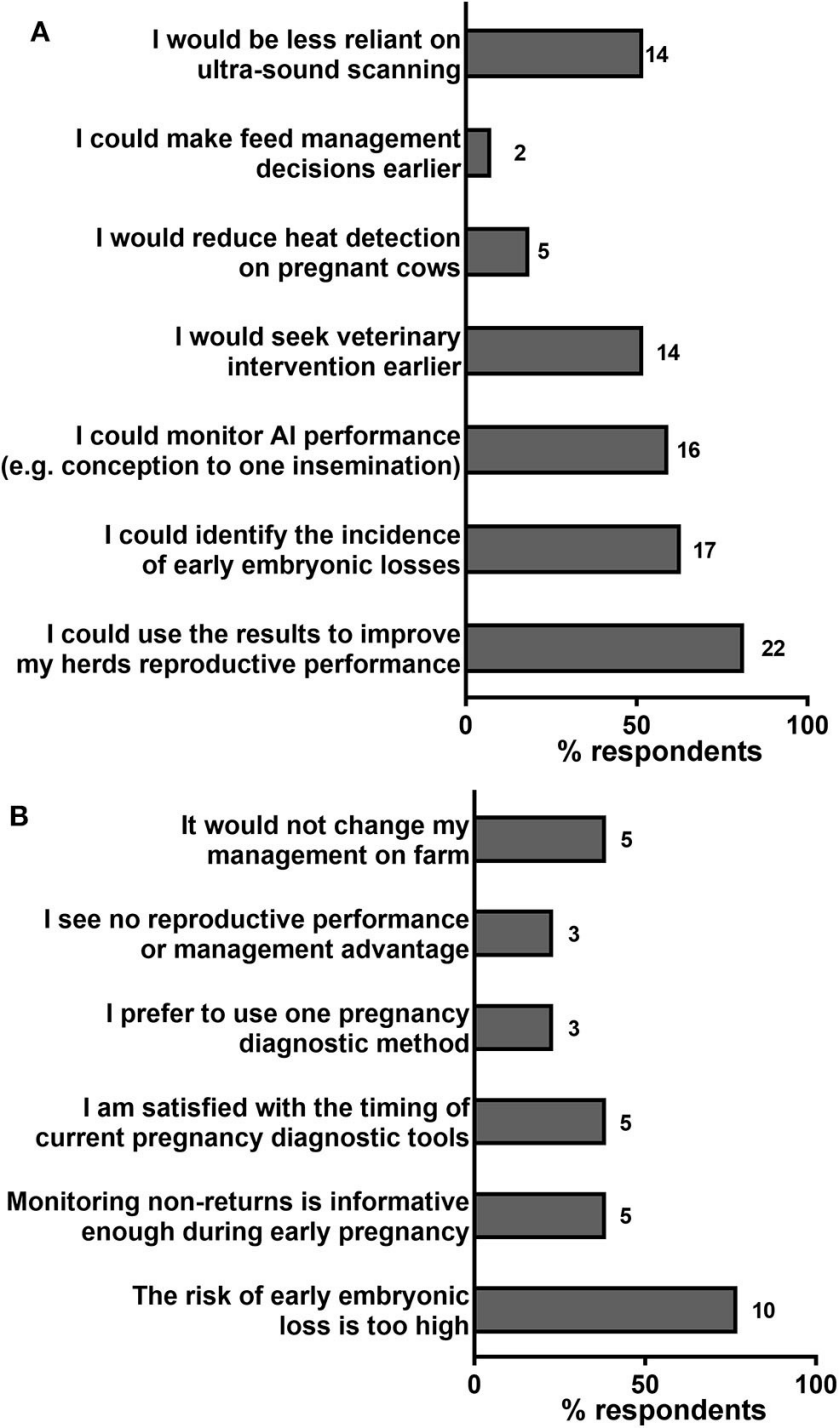
Farmers' responses to why they would use (**A**, *N* = 27) or would be hesitant to use (**B**, *N* = 13) a pregnancy test that could diagnose pregnancy 7–14 days post-insemination. Percentages of respondents selecting each listed statement are shown by horizontal bars. Number of respondents are shown next to each bar.

Farmers were then asked whether or not they would consider using a test that identified pregnancy in cows as early as 7–14 days post-insemination, and a justification was requested for the given response. The majority of farmers (27/40, 67.5%) stated that they would consider using a test that identified pregnancy within the first 2 weeks post-insemination. Within that group, more than half of respondents considered the following advantages in using such approach; “I could use the results to improve my herd's reproductive performance” (22/27, 81.5%), “I could identify the incidence of early embryonic losses” (17/27, 63.0%), “I could monitor AI performance (e.g., conception to one insemination)” (16/27, 59.3%) and “I would seek veterinary intervention earlier” (14/27, 51.9%) (Figure 6A). On the other hand, the most common justification, by large, for not wanting to diagnose pregnancy early was “The risk of early embryonic loss is too high” (10/13, 76.9%) (Figure 6B).

**Figure 7:**
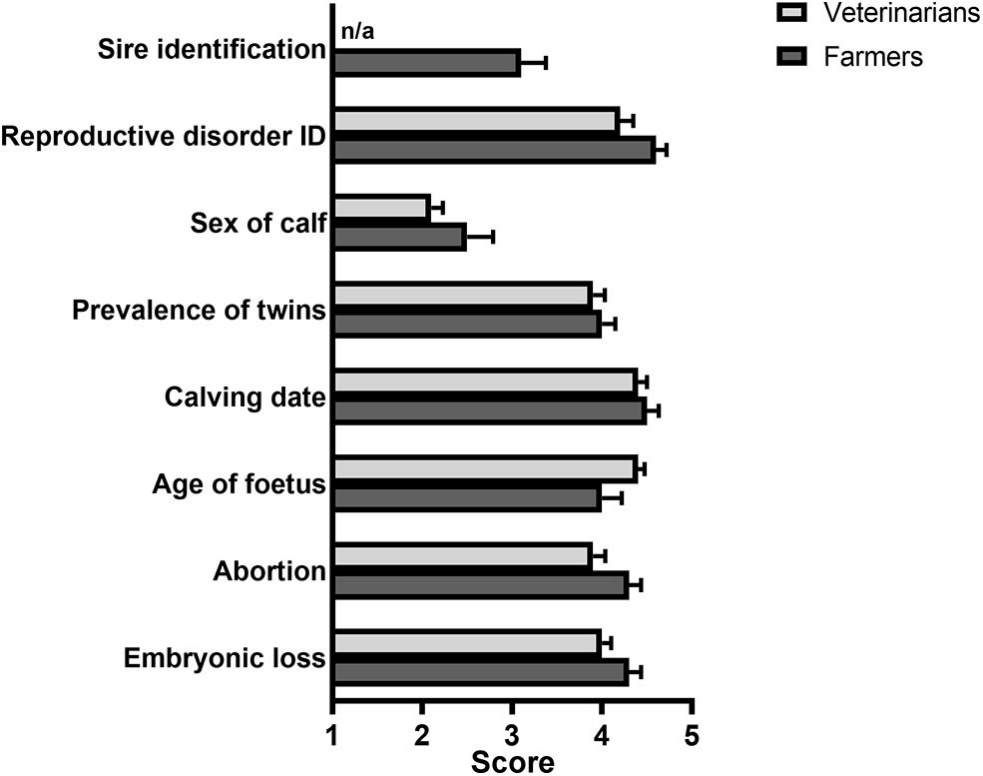
Farmer and veterinarian ratings of the importance of information that can be obtained from pregnancy testing. Respondents were asked to rate their agreement with each piece of information provided from 1 (strongly disagree) to 5 (strongly agree). Mean (± SE) scores are shown. *N* = 40 farmers, 59 veterinarians. The option “Sire identification” was only available in the farmer-tailored questionnaire.

Respondents were also asked to indicate the relative importance of different types of information that may be obtained from a pregnancy test (Figure 7). Particularly, both cohorts were asked to rate: (1) embryonic loss, (2) abortion, (3) age of fetus, (4) calving date, (5) prevalence of twins, (6) sex of calf, and (7) reproductive disorder identification, whereas farmers only were also asked to rate “sire identification.” All types of information were rated as important by both farmers and veterinarians (mean scores ≥3.9), except for sex of the calf (mean ≤ 2.5) and sire identification (mean = 3.1).

As for pregnancy testing, farmers and veterinarians were then asked to rank their preferred sample type for a fertility test, namely, blood, urine or milk. Both groups ranked milk first followed by blood and urine in that order. Finally, they were asked their opinion on what would be an acceptable timeframe for receiving the results of a pregnancy test (Table 7). Most farmers stated that they would prefer to receive the results overnight (14/35, 40.0%) or within 2–3 days (15/35, 42.9%). In contrast, the majority of veterinarians (30/59, 50.8%) stated that they would consider acceptable to report the results on the same day (Table 7).

**Table 7:** Farmers' and veterinarians' opinion regarding the ideal timing to receive the results of pregnancy testing (*N* = 35 farmers and 59 veterinarians).

**Response**	**Cohort**	**Numbers of respondents (*n*)**	**% Respondents**	**% Respondents (95% CI)**
Same day	Farmers	5	14.3	4.8–30.3
	Veterinarians	30	50.8	37.5–64.1
Overnight	Farmers	14	40.0	23.9–57.9
	Veterinarians	13	22.0	12.3–34.7
2–3 days	Farmers	15	42.9	26.3–60.6
	Veterinarians	14	23.7	13.6–36.6
5–7 days	Farmers	1	2.9	0.1–14.9
	Veterinarians	2	3.4	0.4–11.7

## Discussion

The results of this survey provided updated information on current diagnostic practices related to diagnosis of cow fertility and pregnancy in UK-based dairy herds. This information could guide future development of new technologies or improvement of existing ones to increase dairy herd reproductive efficiency and overall productivity.

Participants were primarily selected from an updated list of dairy farmer and veterinary users that was representative of each of the two professional sectors in the UK. Respondent profiles in terms of herd size, calving and feeding systems, and geographical distribution (Figure 1) provided a good representation of the wider UK dairy industry (1). However, the results outlined in this study should be interpreted after considering the following factors that might have introduced some inevitable bias. The number of dairy farmers questioned was small compared to the total number of farms in the UK (13,000). In addition, data was used from 8.0 to 9.8% of all farmers and veterinarians contacted, respectively (after removing submissions containing demographic information only), providing margins of error (90%) of 10.2 and 12.2%, respectively. Altogether, this limits the power of our study and calls for caution before extrapolating these results to the UK-wide dairy industry. Response rates in our survey were slightly below those typically obtained with this type of surveys [10–15%; www.surveygyzmo.com; (12)]. Higher response rates have been reported for postal or interview-based dairy farmer surveys (13, 14), however, online surveys such as ours have distinct advantages, in that they allow distribution of questionnaires to wide target population(s) as well as facilitate reliable processing and analyses of response data, they provide anonymity to participants, and they avoid interviewer bias, among other benefits. Another factor to consider is that our questionnaires were completed by self-selected participants (within our pre-selected list of farmer and veterinarian contacts). Self-selected participants are usually members of the general sample population that are especially concerned about the topic under survey and also have stronger opinions about it (15); they may also be more willing to consider implementing changes (or recommend changes to their clients) in order to improve dairy husbandry and health practices and profitability in the future. Moreover, our questionnaires had to be completed online and were therefore less accessible to participants that do not use internet regularly. The potential for social desirability bias, as described in a recent survey study (16) should also be taken into account. This represents the increased likelihood that participants respond in a manner that will be viewed favorably by others. Finally, although when designing questions for the veterinarian questionnaire all attempts were made to distinguish between “suggested” and “actual” practice in relation to services offered to farmers, answers to some of the questions may have included both types of practices. Moreover, no consideration was given to the specific factors driving the veterinary practices reported in each case. These considerations should also be taken into account when drawing conclusions from this study.

Our results indicated that over 3/4 of UK dairy farmers (77%) use ultrasound, which is believed to provide the most accurate test for diagnosing fertility issues in their herds, and that a large proportion of farms start to fertility-test their cows early after calving (2–4 weeks), with half of those testing the whole herd at that time. Nevertheless, only a small fraction of farmers (15.2%) stated that current fertility tests met their expectations. Based on scores provided (Figure 3), farmers considered cost-effectiveness and, to a lower extent, the ability to detect problems early, as two limitations of existing assays. Taken together, these results indicate that fertility management of dairy herds in the UK could benefit from new or improved diagnostic tests and/or practices that better suited the preferences of farmers.

Results of the pregnancy testing questionnaire revealed that UK dairy farmers overwhelmingly (9 out every 10) choose ultrasonography over alternative tests (progesterone or PAGs) for pregnancy diagnosis. Compared to the latter approaches, ultrasonography (and rectal palpation to some extent) provide a direct approach that is considered to have high diagnostic accuracy and to offer additional information (such as listed in Figure 7) that can aid decision-making by farmers regarding reproductive management of their cows. Yet, similar to fertility testing, <1 in 6 farmers (15.0%) believed that current pregnancy testing meets their expectations, and more than half of the total respondents (52.5%) stated they would pay more for an improved test. Pregnancy testing by transrectal ultrasonography or manual palpation requires regular visits by qualified veterinarian personnel which can cause disruption to farm routine. Moreover, these approaches (as all other listed in Figure 4A) cannot reliably detect pregnancy earlier than 4 weeks after insemination (17, 18). Those are two aspects of current pregnancy testing that farmers indicated they would like to see improved (Figures 5, 6). Interestingly, our study highlighted discrepancies between farmers and veterinarians regarding the need for better diagnostics for dairy cattle. Thus, as was the case for fertility testing, veterinarians had slightly better opinions than farmers about current pregnancy tests although they believed that farmers do not make full use of the tests available, an opinion that is consistent with that expressed by veterinarians in an earlier survey on mastitis and metabolic disease in the UK (19). Moreover, veterinarians also underestimated farmers' dissatisfaction with current tests as well as their willingness to pay more for a pregnancy testing (Table 6). In this regard, consideration needs to be given to the fact that a farmers' stated willingness to pay more is not always indicative of the behavior that will be shown in practice, i.e., when a test is available in the market. These results highlight the importance of better understanding dairy farmers' expectations as end users (e.g., on-farm implementation, accuracy and affordability) and to consider those when designing and developing new diagnostic tools for cattle.

When queried about the possibility of pregnancy-testing earlier than possible with available tests, the majority of farmers (67.5%) would consider advantageous being able to detect pregnancy as early as the 2nd week after insemination. As indicated by some of the respondents (10/40, 25.0%), pregnancy-testing too early carries the risk of falsely diagnosing some animals as pregnant because of a high incidence of embryo loss during the first 2 weeks after insemination (8). However, most farmers considered early testing to offer more advantages than disadvantages, particularly as it could help improve herd performance by providing detailed information on the incidence and timing of embryo loss, and allow prompt re-insemination (at the next oestrus at 21 days) or treatment of infertile cows, as well as reducing dependence on ultrasound. Diagnosing pregnancy in cows within the first 3 weeks after insemination may be feasible in the near future using available technologies, for example quantification of interferon-stimulated genes or pregnancy-associated miRNAs (10, 20, 21).

In summary, this study highlighted current diagnostic approaches related to dairy herd fertility in the UK farms surveyed. Responses from the farmers and veterinarians surveyed identified ultrasound as the primary tool used to detect pregnancy and to identify fertility issues, and highlighted the limited uptake of other available approaches, particularly in regard to pregnancy detection. Farmers also stated that they would use new or improved diagnostic tools that could diagnose pregnancy earlier (before 3 weeks) and identify fertility issues earlier than with current approaches. This information should guide the development of diagnostic tools that meet both the expectations of farmers and veterinarians, and assist in increasing reproductive efficiency and, by extension, milk production, in UK dairy herds. Taking on the opinions and preferences of end users, particularly farmers, early on during diagnostic tool development will be key to ensure their full uptake in a cost efficient manner for increasing dairy herd productivity.

## Data Availability Statement

All datasets generated for this study are included in the article/Supplementary Material.

## Ethics Statement

The studies involving human participants were reviewed and approved by Human Ethical Review Committee at the Royal (Dick) School of Veterinary Studies, University of Edinburgh. The patients/participants provided their written informed consent to participate in this study.

## Author Contributions

FD, NH, MH, TB, and AM designed the study. NH and MH carried our the survey. TT, FD, NH, and CE analyzed the survey data. TT, FD, and CE wrote the manuscript. All authors contributed to the article and approved the submitted version.

## Conflict of Interest

This study aimed to obtain market evidence to support the development of novel diagnostic tests for cattle. NH, MH, and TB are employed by AbacusBio International Ltd. The remaining authors declare that the research was conducted in the absence of any commercial or financial relationships that could be construed as a potential conflict of interest.
