# Supplementary material for: Farmer and Veterinary Practices and Opinions Related to Fertility Testing and Pregnancy Diagnosis of UK Dairy Cows
Source: Front Vet Sci. 2020 Sep 25;7:564209. doi: 10.3389/fvets.2020.564209 (PMC7545959; doi:10.3389/fvets.2020.564209)
Supplement: Supplementary file 1 [file Table_1.DOCX]

**Appendix 1**

**Questions included in the survey tailored for dairy farmer participants:**

**1) Where do you farm?**

- England
- Northern Ireland
- Scotland
- Wales
- Other - Write In (Required)

**2) What is your role?**

- Farm owner/operator
- Farm manager
- Herd manager
- Farm worker/milk harvester

**3) What is your current herd size?**

- <100
- 100-300
- 300-600
- >600

**4) What calving system do you operate?**

- All year round
- Block calving

**5) What farming system do you operate?**

- Total mixed ration
- Grass based
- Combination of total mixed ration and grazing

**6) What method(s) are you currently using for identifying/diagnosing fertility issues?**

- Ultrasound
- Non seen bulling checks
- Body condition score
- Other - Write In (Required):

**7) Tick all that applies when considering the timing of your current fertility testing practices.**

- Whole herd post-calving (2-4 weeks calved)
- Whole herd pre-mating (2-4 weeks pre-mating)
- Whole herd during mating
- Select cows post-calving (2-4 weeks calved)
- Select cows pre-mating (2-4 weeks pre-mating)
- Select cows during mating
- Other - Write In (Required):

**8) How much do you pay per fertility test?**

- <£2.00
- £2.00-£3.00
- £3.00-£4.00
- ≥£5

**9) Rate whether you agree or disagree with the following statements (1=strongly disagree, 5=strongly agree).**

**The methods that I use to identify fertility issues are: (Minimum of three ratings required)**

| Accurate | 1 ____________[__]____________ 5 |
| --- | --- |
| Easy to use | 1 ____________[__]____________ 5 |
| Cost effective | 1 ____________[__]____________ 5 |
| Able to detects issues early | 1 ____________[__]____________ 5 |

**10) If there was a fertility test that met all of your expectations, would you pay more?**

- Yes, I would pay more
- No, I wouldn’t pay more
- My test meets my expectations

**11) Rank the sampling methods from the following list in order that you would prefer for using as a fertility test.**

- Blood test
- Urine test
- Milk test

**12) What is an acceptable time to receive fertility results?**

- Same day
- Overnight
- 2-3 days
- 5-7 days

**13) What method(s) are you currently using for pregnancy detection?**

- Ultrasound
- Rectal palpation
- Progesterone
- Pregnancy associated glycoproteins
- Non-return to service
- Do not pregnancy test
- Other - Write In (Required):

**14) Tick all that applies when considering the timing of your current pregnancy testing practices.**

- Groups/blocks < 20 days post-insemination
- Groups/blocks 30-50 days post-insemination
- Groups/blocks 30-50 days after the end of the mating period
- Individual cows < 20 days post-insemination
- Individual cows 30-50 days post-insemination
- Individual cows 30-50 days after the end of the mating period
- Other - Write In (Required):

**15) How much do you pay per pregnancy test?**

- <£2.00
- £2.00-£3.00
- £3.00-£4.00
- ≥£5

**16) Rate whether you agree or disagree with the following statements (1=strongly disagree, 5=strongly agree).**

**The method/s that I use for pregnancy detection is/are... (Minimum of three ratings required)**

| Accurate | 1 ____________[__]____________ 5 |
| --- | --- |
| Easy to use | 1 ____________[__]____________ 5 |
| Cost effective | 1 ____________[__]____________ 5 |
| Able to detect pregnancy early | 1 ____________[__]____________ 5 |
| Quick at producing results | 1 ____________[__]____________ 5 |
| Not disruptive to my routine | 1 ____________[__]____________ 5 |
| Informative for decision making | 1 ____________[__]____________ 5 |

**17) If there was a pregnancy test that met all of your expectations, would you pay more?**

- Yes, I would pay more
- No, I wouldn’t pay more
- My test meets my expectations

**18) Would you use a test that identified pregnancy in cows as early as 7-14 days post-insemination?**

***Note: pregnancy diagnosis is a yes or no result.***

- Yes
- No

**19) I would use a pregnancy test that could diagnose pregnancy 7-14 days post-insemination because... Tick all that apply.**

***Note: pregnancy diagnosis is a yes or no result***

**[Logic: Hidden unless: #11 Question "Would you use a test that identified pregnancy in cows as early as 7-14 days post-insemination? *Note: pregnancy diagnosis is a yes or no result.*" is exactly equal to ("Yes")]**

- I could use the results to improve my herds reproductive performance
- I could identify the incidence of early embryonic losses
- I could monitor AI performance (e.g. conception to one insemination)
- I would seek veterinary intervention earlier
- I would reduce heat detection on pregnant cows
- I could make feed management decisions earlier
- I would be less reliant on ultra-sound scanning

**20) I would not use a pregnancy detection method that can diagnose pregnancy 7-14 days post-insemination because... Tick all that apply**

***Note: pregnancy diagnosis is a yes or no result.***

**[Logic: Hidden unless: #11 Question "Would you use a test that identified pregnancy in cows as early as 7-14 days post-insemination?
*Note: pregnancy diagnosis is a yes or no result.*" is exactly equal to ("No")]**

- The risk of early embryonic loss is too high
- Monitoring non-returns is informative enough during early pregnancy
- I am satisfied with the timing of current pregnancy diagnostic tools
- I prefer to use one pregnancy diagnostic method
- I see no reproductive performance or management advantage
- It would not change my management on farm

**21) Rate the importance of the following information for making decisions on farm (1=strongly disagree, 5=strongly agree). (Minimum of three ratings required)**

| Embryonic loss | 1 ____________[__]____________ 5 |
| --- | --- |
| Abortion | 1 ____________[__]____________ 5 |
| Age of foetus | 1 ____________[__]____________ 5 |
| Calving date | 1 ____________[__]____________ 5 |
| Prevalence of twins | 1 ____________[__]____________ 5 |
| Sex of calf | 1 ____________[__]____________ 5 |
| Reproductive disorder identification | 1 ____________[__]____________ 5 |
| Sire identification | 1 ____________[__]____________ 5 |

**22) Rank the alternative sampling methods from the following list in the order you would prefer for using as a pregnancy diagnosis.**

- Blood test
- Urine test
- Milk test

**23) What is an acceptable time to receive pregnancy results?**

- Same day
- Overnight
- 2-3 days
- 5-7 days

**Questions included in the survey tailored for veterinarian participants:**

**1) Where do you practice?**

- Scotland
- Wales
- England
- Northern Ireland
- Other - Write In (Required):

**2) What method(s) do you offer for identifying/diagnosing fertility issues?**

- Ultrasound
- Non seen bulling checks
- Body condition score
- Other - Write In (Required):

**3) Tick all that applies when considering the timing of your current fertility testing practices.**

- Whole herd post-calving (2-4 weeks calved)
- Whole herd pre-mating (2-4 weeks pre-mating)
- Whole herd during mating
- Select cows post-calving (2-4 weeks calved)
- Select cows pre-mating (2-4 weeks pre-mating)
- Select cows during mating
- Other - Write In (Required):

**4) On average how much do you charge per fertility test/diagnosis?**

- <£2.00
- £2.00-£3.00
- £3.00-£4.00
- ≥£5

**5) Rate whether you agree or disagree with the following statements (1=strongly disagree, 5=strongly agree).**

**Current fertility testing methods are generally... (Minimum of three ratings required)**

| Accurate | 1 ____________[__]____________ 5 |
| --- | --- |
| Easy to use | 1 ____________[__]____________ 5 |
| Cost effective (to the veterinary business) | 1 ____________[__]____________ 5 |
| Detecting fertility problems early | 1 ____________[__]____________ 5 |
| Frequently used by farmers | 1 ____________[__]____________ 5 |

**6) If there was a fertility test that met all of your expectations, do you think farmers would pay more?**

- Yes, farmers would pay more
- No, farmers would not pay more
- Currently fertility tests meet our expectations

**7) Rank your order of preference for the following methods for fertility testing.**

- Blood test
- Urine test
- Milk test

**8) What is an acceptable time to report fertility testing results?**

- Same day
- Overnight
- 2-3 days
- 5-7 days

**9) What method(s) do you currently offer for pregnancy detection?**

- Ultrasound
- Rectal palpation
- Progesterone
- Pregnancy associated glycoproteins
- Other - Write In (Required):

**10) Tick all that applies when considering the timing of pregnancy testing practices.**

- Groups/blocks <20 days post-insemination
- Groups/blocks 30-50 days post-insemination
- Groups/blocks 30-50 days after the end of the mating period
- Individual cows <20 days post-insemination
- Individual cows 30-50 days post-insemination
- Individual cows 30-50 days after the end of the mating period
- Other - Write In (Required):

**11) How much do you charge per pregnancy test?**

- <£2.00
- £2.00-£3.00
- £3.00-£4.00
- ≥£5

**12) Rate whether you agree or disagree with the following statements (1=strongly disagree, 5=strongly agree).**

**Current pregnancy diagnostic methods are generally... (Minimum of three ratings required)**

| Accurate | 1 ____________[__]____________ 5 |
| --- | --- |
| Easy to use | 1 ____________[__]____________ 5 |
| Cost effective | 1 ____________[__]____________ 5 |
| Able to detect pregnancy early | 1 ____________[__]____________ 5 |
| Producing timely results | 1 ____________[__]____________ 5 |
| Frequently used by farmers | 1 ____________[__]____________ 5 |

**13) If there was a pregnancy test that met all of your expectations, do you think farmers would pay more?**

- Yes, farmers would pay more
- No, farmers wouldn’t pay more
- Current tests meet our expectations

**14) Rate the importance of the following information gained from pregnancy testing (1=strongly disagree, 5=strongly agree). (This question is optional)**

| Embryonic loss | 1 ____________[__]____________ 5 |
| --- | --- |
| Abortion | 1 ____________[__]____________ 5 |
| Age of foetus | 1 ____________[__]____________ 5 |
| Calving date | 1 ____________[__]____________ 5 |
| Prevalence of twins | 1 ____________[__]____________ 5 |
| Sex of calf | 1 ____________[__]____________ 5 |
| Reproductive disorder identification | 1 ____________[__]____________ 5 |

**15) Rank your order of preferance for the following methods for pregnancy diagnosis.**

- Blood test
- Urine test
- Milk test

**16) What is an acceptable time to report pregnancy results?**

- Same day
- Overnight
- 2-3 days
- 5-7 days
